# Effects of *Moringa oleifera* Leaves Extract on High Glucose-Induced Metabolic Changes in HepG2 Cells

**DOI:** 10.3390/biology7030037

**Published:** 2018-06-26

**Authors:** Jorge A. Sosa-Gutiérrez, Mónica A. Valdéz-Solana, Tamara Y. Forbes-Hernández, Claudia I. Avitia-Domínguez, Gonzalo G. Garcia-Vargas, José M. Salas-Pacheco, Oscar Flores-Herrera, Alfredo Téllez-Valencia, Maurizio Battino, Erick Sierra-Campos

**Affiliations:** 1 Facultad de Ciencias Químicas, Universidad Juárez del Estado de Durango Campus Gómez Palacio, Avenida Artículo 123 S/N, Fracc, Filadelfia, 35010 Gómez Palacio, Mexico; sosa_jasg@hotmail.com (J.A.S.-G.); valdezandyval@gmail.com (M.A.V.-S.); 2 Dipartimento di Scienze Cliniche Specialistiche ed Odontostomatologiche (DISCO)-Sez. Biochimica, Facoltà di Medicina, Università Politecnica delle Marche, 60131 Ancona, Italy; tamaraforbe@gmail.com (T.Y.F.-H.); m.a.battino@univpm.it (M.B.); 3 Facultad de Medicina y Nutrición, Universidad Juárez del Estado de Durango Campus Durango, Avenida Universidad y Fanny Anitúa S/N, 34000 Durango, Mexico; avitiaclaudia@gmail.com (C.I.A.-D.); tellezalfredo@gmail.com (A.T.-V.); 4 Facultad de Ciencias de la Salud, Universidad Juárez del Estado de Durango Campus Gómez Palacio, Calzada Palmas 1, Colonia Revolución, 35050 Gómez Palacio, Mexico; ggarcia_vargas@hotmail.com; 5 Instituto de Investigación Científica, Universidad Juárez del Estado de Durango, Avenida Universidad S/N, 34000 Durango, Mexico; jsalas_pacheco@hotmail.com; 6 Departamento de Bioquímica, Facultad de Medicina, Universidad Nacional Autónoma de México, 04510 Ciudad de México, Mexico; oflores@bq.unam.mx

**Keywords:** HepG2 cells, *Moringa oleifera*, mitochondria, UCP2, SIRT3

## Abstract

Mitochondrial dysfunction is a hallmark of diabetes, but the metabolic alterations during early stages of the disease remain unknown. The ability of liver cells to rearrange their metabolism plays an important role in compensating the energy shortage and may provide cell survival. *Moringa oleifera* leaves have been studied for its health properties against diabetes, insulin resistance, and non-alcoholic liver disease. We postulated that *M. oleifera* executes a protective function on mitochondrial functionality in HepG2 treated with high glucose. We evaluated the effect of high glucose treatment on the mitochondrial function of HepG2 cells using a Seahorse extracellular flux analyzer (Agilent, Santa Clara, CA, USA), blue native polyacrylamide gel electrophoresis (BN-PAGE), and western blot analysis. For assessment of mitochondrial abnormalities, we measured the activity of mitochondrial Complex I and IV as well as uncoupling protein 2, and sirtuin 3 protein contents. Our results demonstrate that, under conditions mimicking the hyperglycemia, Complex I activity, UCP2, Complex III and IV subunits content, supercomplex formation, and acetylation levels are modified with respect to the control condition. However, basal oxygen consumption rate was not affected and mitochondrial reactive oxygen species production remained unchanged in all groups. Treatment of HepG2 cells with *M. oleifera* extract significantly increased both protein content and mitochondrial complexes activities. Nonetheless, control cells’ respiratory control ratio (RCR) was 4.37 compared to high glucose treated cells’ RCR of 15.3, and glucose plus *M. oleifera* treated cells’ RCR of 5.2, this indicates high-quality mitochondria and efficient oxidative phosphorylation coupling. Additionally, the state app was not altered between different treatments, suggesting no alteration in respiratory fluxes. These findings enhance understanding of the actions of *M. oleifera* and suggest that the known antidiabetic property of this plant, at least in part, is mediated through modulating the mitochondrial respiratory chain.

## 1. Introduction

A growing body of experimental and epidemiological evidence suggests that *Moringa oleifera* Lam have antidiabetic and antioxidant effects against the harmful damages of oxidative stress and diabetic complications [1,2,3]. The beneficial activities of *M. oleifera* on carbohydrate metabolism have been shown by different physiological processes, including preventing and restoring the integrity and function of pancreatic β-cells, increasing insulin action, improving glucose uptake and utilization [4]. Recent studies have demonstrated that phenolic compounds of *M. oleifera* significantly decreased total intracellular cholesterol, inhibited the activity of HMG CoA reductase (3-hydroxy-3-methyl-glutaryl-coenzyme A reductase), and enhanced low-Density Lipoprotein (LDL) receptor binding activity in HepG2 cells [5]. Furthermore, it was reported that the *M. oleifera* during adipogenesis improves adipocyte functionality and upregulates the expression of uncoupling protein 1 (UCP1), sirtuin 1 (SIRT-1), and peroxisome proliferator-activated receptor gamma coactivator 1-alpha (PGC-1α) involved in thermogenesis modulating lipid metabolism [6]. In association with these results, it has been suggested that mitochondrial proton leak (UCP activity) might play a role in the pathophysiology of diabetic complications [7] and cardiovascular diseases [8]. We recently demonstrated changes in oxygen consumption, supercomplex formation, and increased lipoperoxidation levels in isolated mitochondria from liver of streptozotocin (STZ)-diabetic rats, where *M. oleifera* extract may have a protective role against some of these alteration [9]. These data suggested that *M. oleifera* works intracellularly via several metabolic pathways within mitochondria. Thereby, there are diverse promising candidates for suppressing mitochondrial dysfunction, two potential targets that caught our attention are sirtuins (SIRTs) and uncoupling proteins (UCPs) which seem to be critically important in the pathogenesis of diabetes, cardiovascular disease, and obesity [10].

The SIRTs are a family of nicotinamide adenine dinucleotide (NAD^+^) dependent deacetylases and they play a critical role in restoring homeostasis during stress responses. They can influence multiple protein functions, including DNA—protein interactions, transcriptional activity, subcellular localization, protein stability, and enzymatic activity [11,12]. The crucial role played by SIRTs in the regulation of metabolism has been extensively studied. For example, in liver and heart, SIRT1 regulates gluconeogenic activity by modulating cAMP responsive element binding protein, PGC-1α a nuclear-encoded transcriptional coactivator that regulates the expression of nuclear-encoded mitochondrial proteins, including nuclear respiratory factors 1 and 2 (NRF1 and NRF2), estrogen-related receptor-α (ERR-α), and mitochondrial transcription factor A (TFAM) [13,14]. In addition, SIRT1 activators improve insulin sensitivity in liver and heart. SIRT3 is a member of the sirtuin family that is localized in mitochondria. It is decreased in skeletal muscle of diabetic models [15] and it has been shown that high fat feeding induces a shift in acetylation balance, causing protein hyperacetylation in liver [16], SIRT3 is also involved in the regulation of oxidative phosphorylation through the deacetylation of Complex I and succinate dehydrogenase subunits [17,18]. In diabetes, increases in mitochondrial uncoupling induce mild metabolic stress by dissipating the hydrogen ion gradient across the inner mitochondrial membrane. As a stress response, glucose uptake and NADH oxidation are stimulated with an increase in respiration, NAD^+^ levels, and the NAD^+^/NADH ratio in mitochondria. Thus NAD^+^ dependent SIRT1 is activated [19]. Therefore, SIRT1 and 3 regulation is a promising new therapeutic approach for treating diabetic complications [20] and some research groups are now focusing on the development of high affinity small molecule activators of SIRT1 [21,22].

In this context, it was demonstrated that resveratrol significantly increases SIRT1 activity through allosteric interaction, resulting in the increase of SIRT1 affinity for both NAD^+^ and the acetylated substrate, causing deacetylation of PGC1α, forkhead box protein O1 (FOXO1), and the target of rapamycin kinase 2 (TORC2) which in turn leads to increased fatty acid oxidation and gluconeogenesis [23,24]. However, it is still unknown whether these mitochondrial defects result in change in others SIRTs. 

On the other hand, UCPs are a family of carriers expressed in the mitochondrial inner membrane that uncouple oxygen consumption by the respiratory chain from ATP synthesis. UCP2 is expressed in a wide range of tissues and acts in the protection against oxidative stress, in the negative regulation of insulin secretion by beta cells, and in fatty acid metabolism. Most of the studies about the role of UCP2 in diabetes have focused on the UCP2 functions in β-cells, and the results have shown a deleterious effect of UCP2 in diabetes [25]. As UCP2 is widely expressed in many tissues such as liver, its antioxidant activity makes it logical to search look for benefits on diabetes through counteracting the oxidative stress appeared in diabetes and its complications. Moreover, Korean red ginseng promoted the expression of insulin and downregulated the expression of UCP2 in spontaneously diabetic Goto-Kakizaki rats [26]. Due to the close correlation between these proteins (SIRT and UCP) and mitochondrial oxidative phosphorylation, we hypothesized that *M. oleifera* may exert a protective effect against the development of diabetes through regulatory effect of SIRT3 and UCP2.

Finally, HepG2 cells are a suitable and well characterized model of human liver, which has been widely used in biochemical and nutritional studies [27,28,29]. Numerous studies have used a high concentration of glucose (25 mM) as an in vitro model for investigation of hyperglycemia-induced toxicity which simulated in vivo condition of diabetic ketoacidosis observed in acute or untreated diabetes [30,31,32]. Therefore, we used this cell line to explore the effect of high glucose on modulating mitochondrial SIRT3 and UCP2 and the possible protective role of *M. oleifera* over these alterations to determine the molecular targets by which this plant exerts its beneficial properties in the treatment of diabetes.

## 2. Materials and Methods

### 2.1. Reagents 

All the reagents used in this study were of reagent grade and were purchased from Sigma Aldrich (Toluca, Mexico); Gibco, Thermo Fisher Scientific (Waltham, MA, USA); Invitrogen Thermo Fisher Scientific (Waltham, MA, USA); and Abcam (Cambridge, MA, USA).

### 2.2. Moringa oleifera Extract Preparation

Extract preparation was carried out as previously described [33]. 100 g of crushed dried moringa leaves were macerated for 24 h in 1 L of 20:80 v/v methanol in constant stirring. Solution was then filtered and distilled in a rotary evaporator at 60 °C, the aqueous fraction was then frozen at −80 °C for 24 h prior freeze drying to yield the final powdered extract. The extract was stored at −80 °C until its use.

### 2.3. Cell Culture 

HepG2 cells were grown in 75 cm^2^ flasks in Dulbecco’s modified Eagle’s medium (DMEM) (5 mM glucose) supplemented with 10% fetal bovine serum (FBS) and penicillin/streptomycin and kept at 37 °C with 5% CO_2_. Media was changed every third day until confluence was reached. Then, cells were divided into three groups: control (C); 25 mM glucose (G) and 25 mM glucose + 500 µg/mL of *M. oleifera* extract (GM). Cells were kept in high glucose media for 24 h. After incubation, cells were washed with fresh DMEM and kept in media supplemented with moringa extract for 2 h. Cells were detached with trypsin and resuspended in DMEM to inactivate trypsin, then centrifuged at 600× *g* for 10 min to recover the cellular pellet.

### 2.4. Mitochondrial Isolation 

Mitochondria from HepG2 cells were isolated with mitochondria isolation kit (MITOISO2 Sigma) following manufacturer indications.

### 2.5. Viability Assay 

Ninety-six-well plates were seeded with 5000 cells per well to perform viability experiments. Cells were incubated with different concentrations of moringa extract (50–500 µg/mL), glucose (50 mM), and the combination of both for 24 h at 37 °C with 5% CO_2_. Cells were then incubated for 2 h with 30 µL of 3-(4,5-dimethylthiazol-2-yl)-2,5-diphenyltetrazolium bromide (MTT) solution per well. The media was discarded and 100 µL of dimethyl sulfoxide (DMSO) was used to dissolve the formazan crystals and to extract the blue color. Absorbance was read in a microplate reader at 595 nm. 

### 2.6. Oxygen Consumption Rate 

A measure of 30,000 cells/mL were seeded in 24-well seahorse XF-24 plates and let attach for 24 h. Cells were then incubated with glucose (25 mM) for 24 h, followed by moringa extract addition (500 µg/mL) for 2 h. After incubation, media was replaced with DMEM without FBS and placed at 37 °C without CO_2_. The injection ports were loaded with sequence of oligomycin, carbonyl cyanide 4 (trifluoromethoxy) phenylhydrazone (FCCP), rotenone (Rot), and antimycin A (AA) for final assay concentrations of 2.5 µg/mL, 2.5 µM, 1 µM, and 10 µM, respectively. Flux pack was hydrated overnight and calibrated for 30 min prior oxygen consumption rate (OCR) analysis.

The cellular bioenergetic parameters determined were ATP linked respiration, proton leak, maximal OCR, and reserve capacity. ATP linked respiration was derived from the difference between OCR at baseline and respiration following oligomycin addition. The change in OCR between antimycin A and oligomycin represented the amount of oxygen consumed due to proton leak. Maximal OCR was determined by subtracting the OCR after antimycin A addition from the OCR induced by FCCP. Lastly, the reserve capacity was calculated by the difference between maximal (FCCP) and basal respiration. 

The intermediate turnover state, known as State 3.5 (the state app) and respiratory flux control were derived as detailed in [34]. In these experiments, the assumption is made that State 3 respiration is equivalent to the rate measured after addition of FCCP (State 3_FCCP_ or State 3u) and State 4 is the rate measured after addition of oligomycin (State 4_oligomycin_ or State 4o). These assumptions allowed the calculation of the apparent respiratory state of the cells using the equation

$${\mathrm{State}}_{\mathrm{apparent}}=4-\left[\left(\mathrm{Basal}-\mathrm{Oligo}\right)/\left(\mathrm{FCCP}-\mathrm{Oligo}\right)\right]$$

where Basal represents the basal OCR, Oligo represents the oligomycin-insensitive OCR (proton leak), and FCCP represents the FCCP-stimulated OCR (maximal OCR). Using the same assumptions regarding the relative State 3 and State 4 respiration, the respiratory control ratio (RCR) was calculated as the State 3u rate divided by State 4o rate (maximal OCR/oligomycin insensitive OCR). Coupling efficiency is the proportion of the oxygen consumed to drive ATP synthesis compared with that driving proton leak and was calculated as the fraction of basal mitochondrial OCR used for ATP synthesis (ATP-linked OCR/basal OCR) [35]. Finally, the fraction of respiration that was used under routine conditions to produce ATP (phosphorylating respiration) was estimated as the ratio between ATP-linked OCR and maximal OCR (FCCP) (ATP linked OCR/maximal OCR) as described in [36].

### 2.7. Measurement of Reactive Oxygen Species Levels 

Six-well plates were seeded with 150,000 cells per well and incubated with glucose (25 mM) for 24 h, followed by incubation with moringa extract (500 µg/mL) for 2 h at 37 °C with 5% CO_2_. After incubation, cells were washed with PBS, detached with trypsin and resuspended in a final volume of 0.5 mL. Cell suspension was incubated with 1 µL of CellROX orange reagent for 30 min at 37 °C. The cell suspension was centrifuged at 600× *g* for 10 min and the pellet resuspended in 100 µL of PBS. Measures of 25 µL of the final suspension were used to quantify the fluorescence in a Tali image-based cytometer. 

### 2.8. SDS-PAGE and Western Blot Analysis 

Cellular pellet was resuspended in 0.1 mL of lysis buffer containing 20 mM Tris (tris(hydroxymethyl)aminomethane), pH 7.5, 150 mM KCl, 1 mM ethylenediaminetetraacetic acid (EDTA), and 1% Triton X-100 and protease inhibitor cocktail. Cells were rapidly frozen and thawed three times with liquid nitrogen to ensure maximal cell lysis and centrifuged at 5000× *g* for 15 min, supernatants were recovered and used for further analysis. 50 µg of protein (determined by bicinchoninic acid (BCA) analysis) were loaded into a 12% sodium dodecyl sulfate (SDS)-polyacrylamide gel and run at 120 V for 90 min. Proteins were then transferred to a polyvinylidene difluoride (PVDF) membrane previously activated with methanol. Membranes were incubated overnight with the primary antibody (anti UCP2—ab67241, anti SIRT3—ab189860, anti-acetyl lysine—ab80178, and anti OXPHOS—ab110413) at dilutions of 1:500, followed by 2 h incubation with the secondary antibody at dilutions of 1:1000. The intensity of bands was determined by Image Studio Lite v.5.2 software (LI-COR Biosciences, Lincoln, NE, USA).

### 2.9. BN-PAGE 

Respiratory supercomplexes and complexes were solubilized using digitonin (a very-mild detergent) as described by [37] with minor modifications. Briefly, mitochondrial proteins isolated from HepG2 cells (10 mg/mL) were suspended in 3.5 mL of 50 mM Bis-Tris and 500 mM 6-aminocaproic acid, pH 7.0, and 140 µL digitonin (50% stock) were added to reach a detergent/protein ratio of 2:1 and incubated in this condition during 30 min. The mixture was centrifuged at 100,000× *g* for 30 min at 4 °C and supernatant was recovered.

Supercomplexes and complexes were loaded on a linear polyacrylamide gradient gel (4–10%) for Blue Native PAGE (BN-PAGE). Anode buffer contained 50 mM Bis-Tris/HCl, pH 7.0 and cathode buffer 50 mM tricine, 15 mM Bis-Tris, pH 7.0 and Coomassie Brilliant Blue R-125 dye (0.02%). The voltage was set to 35 V for 10 h at 4 °C and the run was stopped when the sharp line of the dye approached the gel front. Molecular weight of the respiratory complexes and supercomplexes was determined by their electrophoretic mobility and in-gel catalytic activity, using the digitonin-solubilized bovine heart mitochondria as standard. The intensity of bands was determined by Image Studio Lite v.5.2 software.

### 2.10. In-Gel CI and CIV Activities 

The in-gel assays were performed as described by [38] using gel loaded with isolated digitonine-solubilized supercomplexes. CI activity was assayed in a buffer containing 5 mg MTT and 3.75 mg NADH in 10 mL of 10 mM Tris/HCl, pH 7.4. Once activity-staining appeared (10–20 min) reaction was stopped with fixing solution (50% methanol, 10% acetic acid). To assay the activity of complex IV the gel was incubated in 10 mL of 50 mM K_2_HPO_4_, pH 7.2, 10 mg of diaminobenzidine (DAB) and 2 mg of horse heart cytochrome c. After 30–40 min of incubation at 20–25 °C, the activity was observed as a brown precipitate and the reaction was stopped with the fixing solution. The intensity of bands was determined by Image Studio Lite v.5.2 software.

### 2.11. Statistical Analysis 

Data were analyzed by one-way ANOVA using the SigmaPlot v.12.3 (Systat Software, Inc., San Jose, CA, USA). Differences among groups were considered significant when *p* ≤ 0.05.

## 3. Results 

### 3.1. Toxicity of the Extract and High Glucose 

Previous studies have also shown that HepG2 cells are a better model than fresh human hepatocytes to define mitotoxicity [39] and it was reported that high glucose treatment at 25 mM for 72 h increased apoptosis in HepG2 cells through increase oxidative stress [40]. Recent reports demonstrated that the nano-micelle of *M. oleifera* seed oil remarkably induces mitochondrial apoptosis mediating cell death [41]. Moreover, *M. oleifera* aqueous leaf extract treatment resulted in a significant decrease in mitochondrial membrane potential (1 h) and ATP levels (3 h), followed by an increase in (6 h) ROS, caspase activation, proapoptotic proteins expression, and poly [ADP-ribose] polymerase 1 cleavage on different types of cells, including HepG2 [42]. Taken together, both high glucose and *M. oleifera* extract could decrease cell viability by promoting mitochondrial dysfunction and oxidative stress. Therefore, we further investigated whether such experimental conditions affect our cell line.

**Figure 1:**
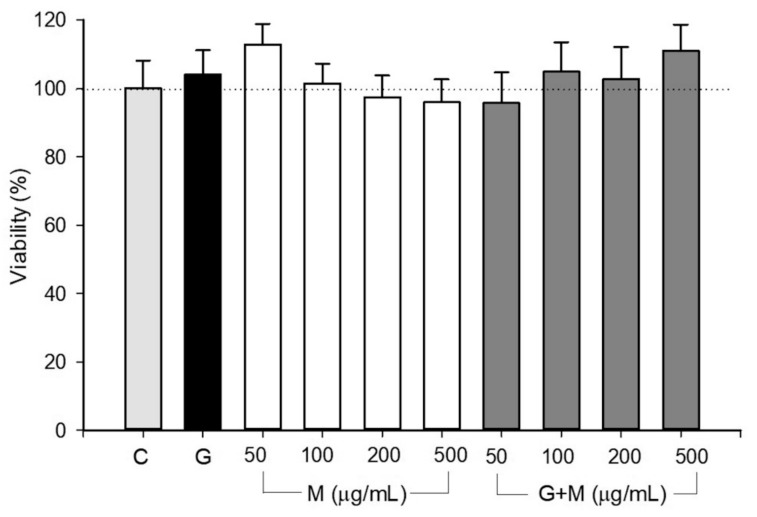
Viability of HepG2 cells treated with glucose and *M. oleifera* extract. Cells were incubated as described in material and methods section and incubated with 25 mM glucose (G) and different concentrations of *M. oleifera* extract (GM; 50–500 µg/mL). Data are expressed as the average ± SD from *n* = 4.

In order to demonstrate that *M. oleifera* components and glucose do not have adverse effects on the overall viability of HepG2 cells, we performed an assay with different concentrations of the extract with or without high glucose. We did not find evidence of apoptosis in glucose treated cells. First, our MTT assay results showed that neither the extract nor glucose have negative effects over the viability of cells. Second, even at larger concentrations (500 µg/mL) *M. oleifera* extract did not showed any difference with respect to control group, in fact, the groups treated with glucose and the extract showed slightly higher viability than control cells (Figure 1). Hence, it is possible that *M. oleifera* extract can induce mitochondrial metabolic changes without affecting cell viability in early stages of treatment, supporting our previous results with an animal model. Although glucose had no negative effects on cells’ viability, it influenced the mitochondrial respiration.

### 3.2. Effect of Moringa Extract on Mitochondrial OCR

To assess the effect of high glucose and *M. oleifera* extract on oxygen consumption rate (OCR) we used the Seahorse XF-24 analyzer. All OCR readings in the three groups were normalized to total protein concentration and the parameters analyzed were; the basal respiration (State II) is controlled by proton leak and substrate oxidation. In the presence of oligomycin, respiration is highly dependent on proton leak. The injection of uncoupling agent FCCP reestablishes electron flux and gives rise to maximum capacity of ATP generation or oxygen consumption. Finally, the residual oxygen consumption can be measured by injection of respiratory inhibitors rotenone/antimycin A. 

**Figure 2:**
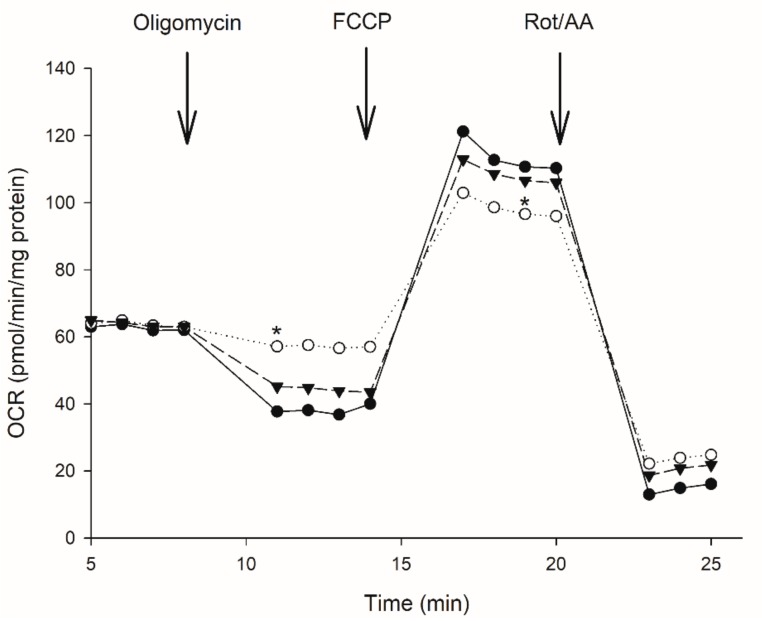
Absolute change in mitochondrial function in intact HepG2 cells treated with glucose 25 mM (white circle) and *M. oleifera* extract 500 µg/mL (inverted gray triangle). The addition of oligomycin at 5 min inhibits ATP production resulting in a decrease in oxygen consumption rate (OCR). The OCR increases in all treatments following the addition of FCCP at 13 min (uncoupled state). Electron transport chain inhibitors mix (Rotenone and Antimycin A) decrease oxygen consumption rates to very low levels inhibiting total mitochondrial respiration at 18 min. * significant difference against control *p* ≤ 0.05.

Initially, the basal respiration did not differ between control and other treatments. HepG2 cells treated with high glucose showed no difference in basal respiration but the results demonstrated that the ATP-linked respiration and respiratory capacity was significantly reduced in the high glucose treated cells in contrast to untreated cells. Oligomycin was added at 7 min to inhibit ATP synthesis, interestingly, we observed that the lack of sensitivity of glucose-treated cells (HG cells) to oligomycin is likely because under these conditions the cells can compensate for mitochondrial impairment by utilizing glycolysis for ATP generation. In contrast, cells grown in high glucose and incubated with *M. oleifera* extract (GM cells) rely mostly on OXPHOS to produce ATP because they are more sensitive to oligomycin. *M. oleifera* treatment not only increased ATP-linked respiration in HG cells but also completely restored their capacity compared to non-treated control cells (Figure 2). The proton ionophore FCCP was added at 13 min to assess the maximum possible oxygen consumption, the FCCP stimulated OCR and demonstrated that uncoupling of OXPHOS provokes an increase in proton leak across the inner mitochondrial membrane and effectively depletes the mitochondrial membrane potential. Interestingly, the stimulation of respiration by FCCP after oligomycin was substantially lower in the presence of high glucose. Moreover, rotenone and antimycin A was added at 19 min to inhibit electron flow through Complex I and III, limiting mitochondrial function with a decrease in OCR. Respiratory control ratio (RCR) was similar between control and GM treatment cells. Control cells RCR was 4.37 compared to HG cells RCR of 15.3, and GM cells RCR of 5.2, these values indicates high-quality mitochondria and efficient oxidative phosphorylation coupling. However, coupling efficiency of control cells was 0.4, which was decreased to 0.1 by the high glucose and significantly suppressed by *M. oleifera* extract (0.31). In addition, phosphorylating respiration in HG cells was 0.065. This was significantly lower that the phosphorylating respiration exhibited by control cells, 0.23. As a comparison, coupling efficiency in control cells was higher than both HG cells and GM cells. Additionally, the state app was not altered between different treatments (Control, 3.56; HG, 3.36, and GM, 3.49), suggesting no alteration in respiratory fluxes which could alter the oxidative phosphorylation machinery.

### 3.3. Uncoupling Protein Level (UCP2)

**Figure 3:**
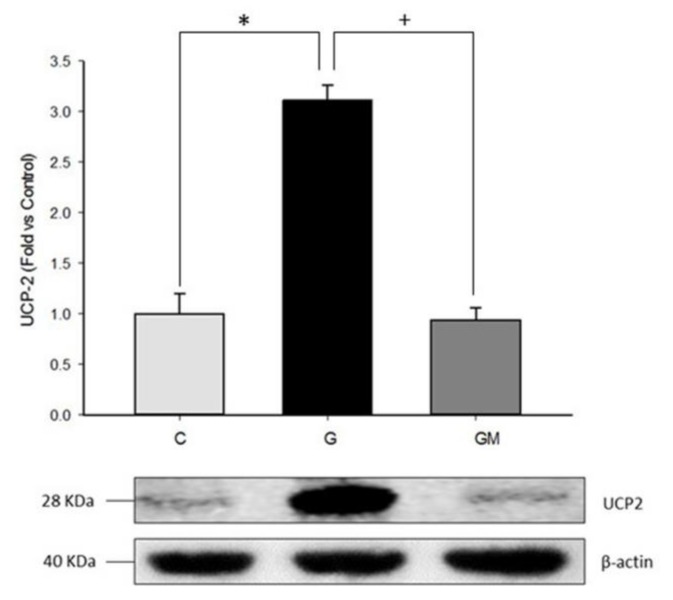
UCP2 protein levels in HepG2 cells treated with glucose 25 mM (G) and *M. oleifera* extract 500 µg/mL (GM). Cells without treatment represent control condition (C). * significant difference against control *p* ≤ 0.05. ^+^ significant difference against G cells *p* ≤ 0.05. Data normalized to control group using β-actin.

To assess whether the above respiration profile data were directly linked to some defect inside the respiratory chain or induce of alternative components, we reasoned that UCP2 activation is likely, at least partially, responsible for the reactive oxygen species inhibitory effect of high glucose in HepG2 cells. In addition, the presence of high glucose caused an increase close to 50% in proton leak (uncoupling protein activity). In support of this hypothesis, we observed that the combined treatment of high glucose and *M. oleifera* extract enhances ATP production and maximal respiration (Figure 2). Our hyperglycemic model showed more than three-fold increase in UCP2 levels in those cells exposed to 24 h of high glucose (Figure 3). We can assume that UCP2 is highly upregulated during hyperglycemia in liver and its presence correlates with the adaptability of hepatic cells to high concentrations of extracellular glucose. Interestingly, after only 2 h of incubation with *M. oleifera* extract, UCP2 levels normalized (Figure 3). These results suggest that antioxidant properties of extract regulate the UCP2 expression in HepG2 and possibly the ROS production.

### 3.4. Reactive Oxygen Species Levels

Higher production of ROS is often stated as both cause and consequence of mitochondrial dysfunction [43]. There is evidence that supports the fact that under hyperglycemic conditions ROS production is significantly higher [44]. To identify the role of ROS in the context of hyperglycemia and mitochondrial dysfunction, we carried out fluorescence tests to HG cells and GM cells. However, after performing CellROX orange fluorescence test, data from the Tali cytometer showed no significant changes of ROS between control cells (5 ± 1.39% of cells) and those treated with glucose (4 ± 1.42%), nor the combination glucose plus *M. oleifera* (4 ± 1.42%). Therefore, we performed repeated tests using Amplex Red ROS-specific probe, and all data were negative for high glucose induced ROS production (data not shown). Hence, the lack of ROS overproduction in our model suggests that diverse metabolic adjustments have occurred at the level of the OXPHOS proteins, which allows cells to adapt to the high glucose environment, and that some regulatory mechanisms could be playing an important role. 

### 3.5. OXPHOS Activities

**Figure 4:**
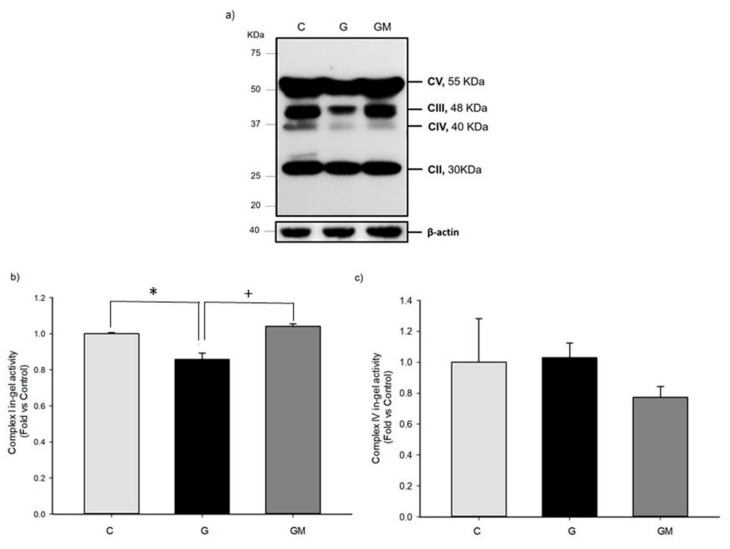
(**a**) Characterization of OXPHOS proteins expressed in HepG2 cells during high glucose with or without *M. oleifera* extract. OXPHOS cocktail specificity demonstrated by Western blot of MTC01 subunit of CIV, SDHB subunit of CII, UQCRC2 subunit of CIII, NDUFB8 subunit of CI, and ATP5A subunit of CV. BN-PAGE. Activity of CI (**b**) and CIV (**c**) in isolated mitochondria from HepG2 cells under normal (C), glucose 25 mM (G), and 500 µg/mL *M. oleifera* extract (GM) conditions. * significant difference against control *p* ≤ 0.05. ^+^ significant difference against G cells *p* ≤ 0.05. Data normalized to control group using β-actin.

In order to evaluate this possibility, we measured both the specific activities and analyzed the expression level of individual respiratory complexes. Initially, we evaluated the activity of Complex I and IV to establish if respiration disruptions observed during hyperglycemia are consequence of protein activities. Interestingly, Complex I activity was significantly lower in those HG cells (Figure 4b), correlating with the disruptions observed in respiratory rates in the same samples (Figure 2). As for Complex IV, we found no significant differences among groups (Figure 4c). Despite the clear effect of high glucose over Complex IV protein levels, this protein can modulate its activity to adapt and sustain cell viability. We were not able to measure Complex III activity, however, due to the lower levels of Complex III and the diminished activity of Complex I found in HG cells, we can assume that these two complexes are responsible for the respiratory alterations observed in our hyperglycemic model. As shown in Figure 4a, Complex III significantly decreases when the cells are treated with high glucose, interestingly this effect is reverted after only 2 h of incubation with 500 µg/mL of *M. oleifera* extract. On the other hand, Complex IV (Figure 4a) is also decreased in those cells incubated with high glucose, but moringa extract can revert this negative effect. Although protein levels are not as high as control group, these are still significantly different from those found in HG cells. This finding is consistent with [45], who used mass spectrometry analysis to report a 46% decrease in Complex III levels alongside with 20–30% downregulation of subunits from Complex I–IV in cardiac and skeletal muscle mitochondria from diabetic rats. In other reports, proteomic analysis of obese and type 2 diabetic skeletal muscle mitochondria, found less presence of several mitochondrial proteins, including those in the electron transport chain in both human [46] and mice samples [47]. This suggests that tissues with high energy demand or involved in glucose homeostasis are the first ones to suffer from alterations in OXPHOS subunits under hyperglycemic conditions.

### 3.6. Mitochondrial Supercomplex Levels

Recent experimental evidence has replaced the random diffusion model of electron transfer with a model of supramolecular organization based upon specific interaction between individual respiratory complexes [48]. Supercomplexes (SC) is the term given to associations between different mitochondrial complexes [49]. Three of the electron-transfer complexes form SC and several functions have been attributed to SC in mitochondria, and although information regarding this matter is controversial, there are reports of SC modulating ROS formation and facilitate efficient energy generation [50,51]. 

**Figure 5:**
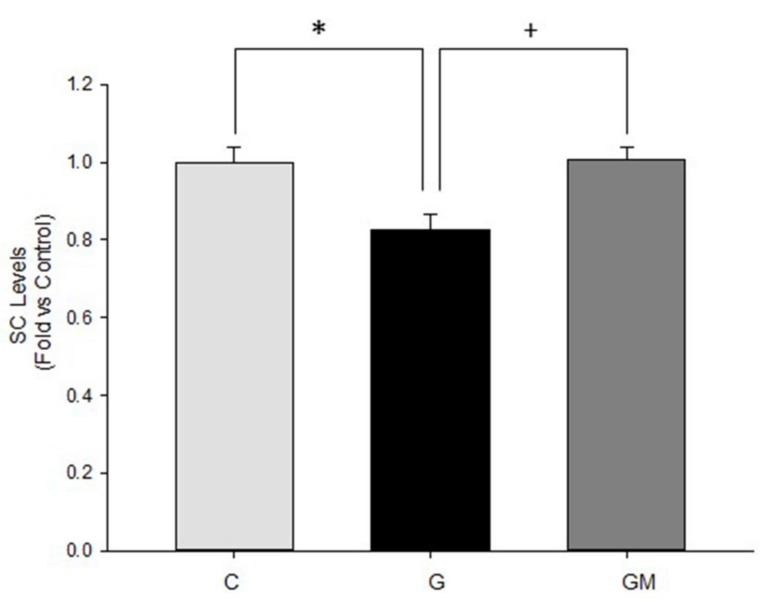
Interactions between mitochondrial complexes in HepG2 cells under normal (C), glucose 25 mM (G), and 500 µg/mL *M. oleifera* extract (GM) conditions. * significant difference against control *p* ≤ 0.05. ^+^ significant difference against G cells *p* ≤ 0.05.

Since we observed changes in protein levels from Complexes III and IV, which are important components of SC, we conducted experiments using BN-PAGE to assess the amount of SC in our model to establish if complex interaction is lost under hyperglycemic conditions. Same as in individual complexes, exposition to high glucose lowers the overall interaction between mitochondrial complexes. Again, the presence of *M. oleifera* extract reverted this effect (Figure 5) indicating that components in the extract are not only capable of preserving OXPHOS protein levels, but also preserving the interactions between them, ensuring a better mitochondrial metabolism. 

### 3.7. Acetylation Levels by SIRT3

Protein post-translational modification is an important process for quickly and transiently modifying the structure of a protein by the covalent addition of functional groups, proteolytic cleavage of regulatory subunits, or degradation of entire proteins, which causes changes in enzyme activity as well as interfering or aiding protein-protein interactions. To date, no work has specifically analyzed the role of post-translational modification in SC assembly or function, but there is a large body of information on post-translational modification of individual respiratory complexes and other mitochondrial proteins [52]. 

**Figure 6:**
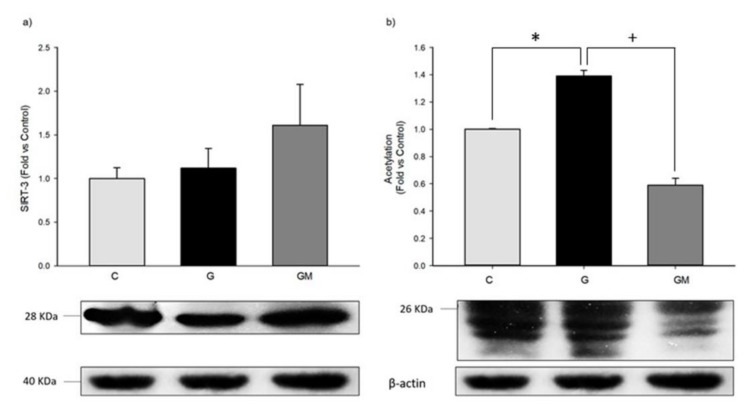
Sirt3 (**a**) and overall acetylation (**b**) protein levels in HepG2 cells under normal (C), 25 mM glucose (G) and 500 µg/mL *M. oleifera* extract (GM) conditions. * significant difference against control *p* ≤ 0.05. ^+^ significant difference against G cells *p* ≤ 0.05. Data normalized to control group using β-actin.

Our results indicated that acetylation percentage is higher in those cells treated with high glucose, and after 2 h of exposure to *M. oleifera* extract, acetylation levels diminished significantly, even below control levels (Figure 6a). Our results regarding SIRT3, showed that there is no significant difference among groups, however, levels in *M. oleifera* treated cells are slightly higher than those found in high glucose treated cells (Figure 6a), existing the possibility that lower acetylation levels in cells treated with the extract are due to higher amounts of SIRT3, and of course, other SIRTs. Our data is contrasting with previous findings where the content of SIRT3 was heavily decreased in diabetic pancreas and lung [53], possibly the alterations in SIRT3 in diabetes are likely tissue dependent [54]. Moreover, when total mitochondrial protein acetylation profile was assessed by anti-acetylation western blot analysis, an increased acetylation on numerous proteins could be detected in diabetes [55], which corresponds with our results (Figure 6b). 

Although we were not able to identify the acetylated mitochondrial proteins, our results clearly show that the level of acetylation in the high glucose treated cells was increased by 40% with respect to the control group. In addition, the acetylation levels were decreased three times with respect to the group treated with high glucose plus *M. oleifera* (Figure 6b). It is difficult to determine which biochemical feature is affected by acetylation; enzyme activity, protein-protein interactions, protein–DNA interactions, stability, localization, allostery, and others. 

## 4. Discussion

There is significant evidence that energy production is impaired during diabetes; however, the molecular events involved are poorly understood. Many compounds isolated from *M. oleifera* have been reported to show antidiabetic and biological properties [56,57]. However, the molecular targets in which the phytochemicals of the *M. oleifera* extract act are unknown. In addition, a recent interest has been devoted to studying the effects on mitochondria of natural compounds as quercetin, resveratrol, and curcumin [58]. Many of these compounds turned out to exert their functions by affecting mitochondrial function, either directly, by inhibiting specific enzymes, or indirectly, by modulating signal from or to mitochondria [59,60,61].

Reports in C57BL/6 mice, showed that quercetin clearly reduced the expression levels of mitochondrial proteins that control mitochondrial dynamics. Interestingly, they found that quercetin reduced the activity only in monomeric Complex IV [62]. Our previous findings with liver isolated mitochondria of STZ-treated rats demonstrated changes in both amount and composition of SC [9], which were replicated in HepG2 cells. Our results show that high glucose also reduced the levels of OXPHOS proteins subunits from respiratory complexes (Figure 4a). Specifically, UQCRC2 subunit of Complex III and MTC01 subunit of Complex IV were significantly reduced. While SDHB subunit of Complex II, and ATP5A subunit of Complex V were not decreased in high glucose treatment. Interestingly, we were not able to detect the representative band of the NDUFB8 subunit of Complex I in either control cells or those treated with high glucose and/or *M. oleifera* extract. However, all groups present significant specific enzymatic activity of this complex. One possible explanation is that the antibody recognition region into the Complex I protein subunit is not available because this complex is always associated with other components of the respiratory chain. In addition, the inhibition of Complex I in high glucose treated cells would cause a decrease in energy supply that would in turn lead to a higher AMP/ATP ratio, and the concomitant activation of AMP-activated protein kinase, although an increase in UCP2 levels could also produce the same result [63]. These results support the idea of several groups who have proposed a stabilizing factor for respiratory supercomplex assembly, cytochrome c oxidase (COX) subunit 7a-related polypeptide (COX7RP) [64,65]. Recently, it has been shown that a metabolic phenotype of Cox7rp knockout mice exhibit lower blood glucose levels after insulin or pyruvate injection. Notably, ATP synthesis rate was reduced in Cox7rp knockout mice liver, in accordance with decreased percentages of Complex III subunit RISP and Complex COX1 involved in respirasome fraction [65]. This result suggests that COX7RP-mediated mitochondrial respiration plays crucial roles in the regulation of glucose homeostasis and its impairment will lead to the pathophysiology of metabolic states.

On the other hand, computational studies have shown that up to 20% of mitochondrial proteins can be acetylated on their lysine residues and are putatively regulated by SIRTs [43]. Three NAD-dependent deacetylases; SIRT3–5; are localized to the mammalian mitochondria. SIRT3 has been implicated in regulating metabolism by deacetylating Complex I subunit NDUFA9 and affect NADH-dependent respiration in mice, as well as α and OSCP subunits from F0F1ATPase and the SdhA subunit from Complex II, demonstrating the role of acetylation/deacetylation in the regulation of oxidative phosphorylation [17,18]. However, from the 700 acetylated mitochondrial proteins, only 26 proteins that display functional effects when acetylated [66]. Several acetylome proteomic studies have identified many lysine-acetylated mitochondrial proteins, including six tricarboxylic acid (TCA) cycle proteins, 26 proteins involved in oxidative phosphorylation, 27 β-oxidation, 8 associated with amino acid metabolism, 10 with carbohydrate metabolism, 3 with nucleotide metabolism, and 2 with the urea cycle [67,68]. With respect to mitochondrial dehydrogenases, 21 were lysine acetylated, among them, 9 subunits of NADH dehydrogenase (Complex I). In this study, Figure 6b shows also that mean value of acetylation of high-glucose treated cells was significantly (at *p* < 0.05) increased compared with control cells. Treating these high-glucose treated cells with *M. oleifera* extract significant (at *p* < 0.05) ameliorated with that high-glucose treated cells. In contrast, Figure 6a shows also that the mean value of SIRT3 was not significantly increased due to high glucose treated cells. Interestingly, treating these cells with *M. oleifera* extract no significantly reduced the SIRT3. These data suggest that other members of SIRTs family may be participate into mitochondria as SIRT1 or 5. However, it is necessary more experimental evidence.

Increased oxidative stress has been hypothesized to activate uncoupling protein 2 (UCP2) which is a regulator of ROS production in the inner membrane of mitochondria. In addition, the liver is the largest metabolic organ in the human body, and mitochondrial proton leak accounts for 20–30% of the oxygen consumption of isolated resting hepatocytes [69]. In the liver, UCP2 has been localized to Kupffer cells, with very low or undetectable levels in hepatocytes. However, this expression pattern appears to be the opposite in fatty liver [70]. Accordingly, we found that the levels of UCP2 was increased in HG cells (Figure 3) and *M. oleifera* extract caused remarkable decrease in UCP2 expression, this last finding is probably due to a direct modulation of mitochondrial environment by components in the extract, where UCP2 is no longer needed in higher levels. This is supported by the fact that *M. oleifera* extract is also capable of restoring alterations in mitochondrial free fatty acids accumulation, a known activator of UCP2 [71,72,73,74], and previous reports where UCP2 levels are regulated by quercetin [74], a component present in our extract [33]. These results suggest that HepG2 protection against high glucose injury is associated with the upregulation of UCP2. Thus, our results are in accordance with findings of non-alcoholic fatty liver disease (NAFLD) [75]. 

NAFLD is part of the metabolic syndrome with insulin resistance as a primary underlying derangement. Available evidence suggests that UCP2 may theoretically contribute to pathogenesis of NAFLD [76]. In addition, the expression of SIRT1 is significantly lowered and UCP2 increased in the liver of rats with diabetes and NAFLD. It was proposed that UCP2 regulates the activity of SIRT3 through sensing the energy levels and, in turn, maintaining the mitochondrial steady state, which demonstrates a cytoprotective effect on cerebral ischemia-reperfusion injury [77]. UCP2 induces mitochondrial proton leak and increases susceptibility of non-alcoholic steatohepatitis liver (NASH) to ischemia-reperfusion injury [78]. Moreover, cardiolipin a phospholipid located at inner mitochondrial membrane, plays an important role in several processes involved in mitochondrial bioenergetics and apoptosis. Cardiolipin peroxidation has been associated with the destabilization of mitochondrial respiratory supercomplexes could be another factor contributing to ROS generation and to mitochondrial bioenergetic decay in NAFLD [79]. 

On the other hand, metformin can increase the expression of SIRT1 and reduce the expression of UCP2, with negative correlation between the expression of SIRT1 and UCP2 [80]. Berberine can downregulate the expression level of UCP2 mRNA and UCP2 proteins of hepatic tissue from NAFLD rats [81]. Interestingly, dietary polyphenols have been identified to offer a potential therapy for NAFLD and its progression to nonalcoholic steatohepatitis [82]. Several polyphenols, such as kaempferol, have been reported to activate both SIRT1 and SIRT3 [83]. Polyphenols found in beverages, such as red wine and grape juice, could bear an effect on energy metabolism, being able to increase UCP2 expression, by increasing energy expenditure, even when administered in a high fat diet situation [84]. Thus, in line with our results, decreased expression of the mitochondrial biogenesis transcription factors PGC1α and NRF1, and decreased expression of respiratory Complex I and V subunits NDUFS8 and ATP5G1 in the HepG2 cell model of steatosis have been reported. The treatment with different polyphenols protected by more than 50% against the oleic acid induced increase in ROS and prevented the decrease of UCP2 [85]. 

**Figure 7:**
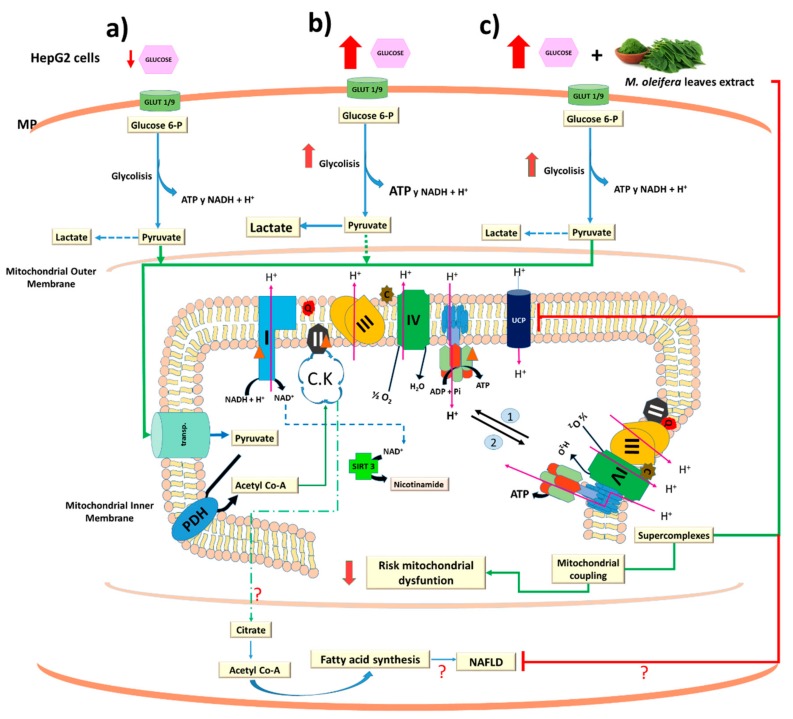
Proposed model for uncoupling protein 2-acetylation by sirtuins signaling pathway on HepG2 treated with high glucose and *M. oleifera* extract. The solid lines indicate carbon skeletal main flux to mitochondria (**a**). While that punted lines indicates the secondary pathways. PDH, pyruvate dehydrogenase; CK, Krebs cycle. The numbers 1 and 2 indicates the dissociation and association of the mitochondrial supercomplexes. Red and green lines indicate inhibition and activation of metabolic process by high glucose (**b**) and *M. oleifera* extract (**c**), respectively. Glut 1 and Glut 9 as major contributors to glucose influx in HepG2 cells [87]. MP, plasma membrane. Red triangle indicates potential site of acetylation in mitochondrial complexes.

Taken together, we proposed a model to explain our data. Under physiological conditions (Figure 7a), mitochondria play a key role in energy metabolism by generating most of the energy used by mammalian cells. The redox power from organic acids oxidation is provided to the respiratory chain by reduced donors (NADH + H^+^; FADH_2_) or directly by specific dehydrogenases via electron-transfer complexes to the quinone pool before reducing final electron acceptor (molecular oxygen). Electron flow is conveyed along the mitochondrial respiratory chain and part of its energy is converted to an electrochemical force by pumping out protons across the inner mitochondrial membrane. This generates electrochemical gradient (also called the proton motive force, pmf) that can be used to synthesize ATP or exchange proteins or ions (Ca^2+^) across the inner mitochondrial membrane. The efficiency with which reduced equivalents are used to generate ATP by oxidative phosphorylation is dependent on mitochondrial coupling. Uncoupling the proton transport across the membrane participates in the regulation of energy homeostasis and defaults in electron transfer can enhance ROS production. Under conditions of excess in energy intake (high glucose) and tight mitochondrial coupling (Figure 7b), pmf can rise to a maximum. Thus, mitochondrial respiratory complexes are highly reduced and may release electrons directly to oxygen resulting in a higher ROS production that could alter oxidative phosphorylation system and lead to a drop of mitochondrial ATP levels. Thus, excessive ROS production would lead to induced uncoupling protein 2 (UCP2). UCP2 is thought to protect against oxidative stress although, alternatively, it could play an energy dissipation role. Consequently, in our study, the increase of UCP2 associated with the increase of proton leak (uncoupling state) may improve the mitochondrial NAD^+^/NADH ratio by suppressing the ATP synthesis and ROS production. SIRT3 is a key regulatory protein, which can sense the NAD^+^ levels. Therefore, it might be possible that UCP2 increases the NAD^+^/NADH ratio to activate SIRTs in high glucose conditions. Furthermore, treatment of high glucose treated cells with *M. oleifera* extract (Figure 7c), increases the levels of acetylation and lowers the UCP2 protein. *M. oleifera* also increases the levels of supercomplexes to optimize the Complex I activity and coupling state. In accordance with these results, we also observed a recovery of the disturbed bioenergetics homeostasis (RCR, coupling efficiency and phosphorylating respiration). Finally, when energy stores are plentiful, Krebs cycle intermediates accumulate, and citrate is transported back into cytoplasm where is converted to acetyl-CoA, which is the first step of endogenous fatty acid synthesis and lipogenesis is a central abnormality in NAFLD (Figure 7 and [86]. However, this last metabolic alteration requires further investigation. 

In conclusion, *M. oleifera* treatment significantly reduced acetylation of mitochondrial proteins and subsequent increase in amount of supercomplexes and Complex I activity, while it dramatically decreased UCP2 expression in high glucose treated HepG2 cells. *M. oleifera* could be a potential source of mitochondrial drugs for diabetes and NAFLD.
